# Electroacupuncture Improves Neurobehavioral Function Through Targeting of SOX2-Mediated Axonal Regeneration by MicroRNA-132 After Ischemic Stroke

**DOI:** 10.3389/fnmol.2018.00471

**Published:** 2018-12-20

**Authors:** Xiaoying Zhao, Fuhai Bai, Erfei Zhang, Dandan Zhou, Tao Jiang, Heng Zhou, Qiang Wang

**Affiliations:** ^1^Department of Anesthesiology and Perioperative Medicine, Xijing Hospital, Air Force Medical University, Xi’an, China; ^2^Department of Anesthesiology, Second Hospital of Shanxi Medical University, Taiyuan, China; ^3^Department of Anesthesiology, The Affiliated Hospital of Yan’an University, Yan’an, China; ^4^Department of Anesthesiology, The Northwest Women’s and Children’s Hospital, Xi’an, China; ^5^Department of Anesthesiology, The First Affiliated Hospital of Xi’an Jiaotong University, Xi’an, China

**Keywords:** stroke, electroacupuncture, microRNA-132, SOX2, neurite outgrowth

## Abstract

Our previous studies have shown that electroacupuncture (EA) enhances neurobehavioral functional recovery after ischemic stroke, however, the underlying regulatory mechanisms remain unclear. MicroRNAs (miRNAs) are abundant in the brain and are involved in post-transcriptional gene regulation. During cerebral ischemia reperfusion, miRNAs perform numerous biological functions in the central nervous system related to regeneration and repair of damaged nerves. Our previous studies also have shown that the expression of miRNA-132 (miR-132) is obviously down-regulated after stroke by middle cerebral artery occlusion (MCAO), which can be up-regulated by EA. This study aimed to identify whether up-regulation of miR-132 by EA improved the damaged nerves after stroke and to screen the potential target of miR-132. The results showed that EA up-regulated miR-132 thus suppressing SOX2 expression *in vivo* after MCAO, which obviously ameliorated neurobehavioral functional recovery. Moreover, our results also suggested that up-regulated miR-132 suppressed SOX2 in primary neurons after oxygen-glucose deprivation (OGD), which promoted neurite outgrowth. In conclusion, EA enhances neurobehavioral functional recovery against ischemic stroke through targeting of SOX2-mediated axonal regeneration by miR-132.

## Introduction

A leading cause of disability in western countries is stroke, which has high lethality. On average, every 40 s someone in the United States has a stroke (Scherbakov et al., 2013; Benjamin et al., 2017). This disability is primarily attributed to the brain lesion, which may result in permanent neurological deficits. However, there is no single effective recovery aid available for the rehabilitation of patients after stroke (Lindau et al., 2014). Acupuncture, which is an important part of traditional Chinese medicine, promotes the recovery of neurological function, and thus improves the quality of life after stroke (Cao et al., 2016; Jiang et al., 2016; Yang et al., 2017). Our previous studies have shown that pretreatment with electroacupuncture (EA), from Chinese traditional acupuncture and modern electrotherapy, has neuroprotective effects on preventing the brain from ischemia-reperfusion(I/R) injury (Xiong et al., 2003; Wang et al., 2005). But the underlying regulatory mechanisms of EA in stroke remain unclear.

MicroRNAs (MiRNAs) are small, non-coding transcripts which regulate the expression of target genes and provide a crucial and pervasive layer of post-transcriptional gene regulation. MiRNAs are abundant in the nervous system and are likely to be important mediators of neuronal plasticity (Kosik, 2006; Fiorenza and Barco, 2016; Wang and Bao, 2017). Numerous studies have demonstrated that miRNAs play an important role in neuroprotection. For example, miR-132 and miR-212 are tandem miRNAs whose expression is necessary for the proper development, maturation and function of neurons, and their deregulation is associated with several neurological disorders (Wanet et al., 2012; Bicker et al., 2014). Our previous study showed significant changes in 20 differential miRNAs, including miR-132, in the ischemic penumbra of rats by microarray analysis, and miR-132 expression was markedly down-regulated after the middle cerebral artery occlusion(MCAO)injury. Additionally, EA treatment up-regulated the expression of miR-132 (Deng et al., 2016). Therefore, this study examined the function and mechanism of miR-132 in EA treatment-induced neuroprotection after stroke.

MicroRNAs are involved the expression of target genes and proteins networks through Watson-Crick base pairing. The sex determining region Y-box 2, also known as SOX2, is one of the key transcription factors involved in the maintenance of neural progenitor identity. SOX2 expression level must be tightly controlled for proper neural development and differentiation (Amador-Arjona et al., 2015; Albright et al., 2016; Fujita et al., 2016). Additionally, SOX2 plays a role during neurogenesis as well as maturation of primary neurons (Nishimura et al., 2017), and different levels of SOX2 exert different effects on neurogenesis and maturation (Klajn et al., 2014). Suppression of SOX2 activity leads to premature cell cycle exit and initiation of neuronal differentiation (Bylund et al., 2003; Graham et al., 2003). SOX2 facilitates the repair of injured cortical neurons (Heinrich et al., 2014). We found that SOX2 was a potential target of miR-132 by miRNA database. However, whether miR-132 could enhance neurobehavioral functional recovery by regulating the expression of SOX2 remains unknown.

Hence, the present study aimed to determine the pivotal role of miR-132 in EA-related rehabilitation of ischemic stroke. Our study is the first to reveal that miR-132 may regulate the expression of SOX2, which provides a new mechanism of EA treatment after stroke.

## Materials and Methods

### Animals

Male Sprague-Dawley rats (SD) (weighing 280–300 g) (provided by the Experimental Animal Center of the Fourth Military Medical University) were housed under controlled conditions with a 12-h light/dark cycle, temperature of 21 ± 2°C and humidity of 60–70% and allowed free access to standard rodent food and water. The experimental protocol was approved by the Ethics Committee for Animal Experimentation of the Fourth Military Medical University and all experiments were performed according to the Guidelines for Animal Experimentation of the Fourth Military Medical University (Xi’an, Shaanxi province, China).

### Middle Cerebral Artery Occlusion (MCAO) Model

The focal cerebral ischemia was established in rats by MCAO as previously described (Zhao et al., 2015). Briefly, after intraperitoneal anesthetization with 10% chloral hydrate (350 mg/kg), the right middle cerebral artery was occluded for 90 min using a 3-0 nylon monofilament suture (Ethicon Nylon Suture, Ethicon Inc., Sukagawa, Japan). Then the suture was removed to allow subsequent reperfusion. After recovery from anesthesia, rats were returned to their home cages. During surgery, cerebral blood flow was monitored by a transcranial laser Doppler flowmetry (PeriFlux 5000, Perimed AB, Sweden) and the rectal temperature was monitored and maintained at 37.0–37.5°C by surface heating or cooling (Spacelabs Medical Inc.). For the sham group, identical operation was performed without inserting the suture.

### EA Treatment

At 24 h after MCAO, the rats were stimulated with the HWATO electronic acupuncture treatment instrument (model No. SDZ-V, Suzhou Medical Appliances Co., Ltd., Suzhou, China) at the Baihui (GV 20) acupoint (Zhao et al., 2015) with an intensity of 1–2 mA and dense-disperse frequency of 2/10 Hz. All the rats received EA treatment for 30 min/day for five consecutive days, the core body temperature was maintained at 37.0 ± 0.5 °C by surface heating or cooling.

### Intracerebroventricular Injection and Cell Transfection

Rats were intraperitoneally anesthetized with 10% chloral hydrate (350 mg/kg) and placed in a stereotaxic frame. A 26-gauge brain infusion cannula was stereotaxically placed into the right lateral ventricle (-0.8 mm posterior to the bregma; 1.5 mm lateral, and 2.6 mm below the skull surface) and fixed with dental gel. MiR-132 mimic (1 pmol/g body weight in 2 μL) (Tao et al., 2015), inhibitor or negative control were mixed with Lipofectamine 3000 (4 μL; 6 μL total volume) and incubated for 25 min before injection. The liposomal coated transfection solution was injected at 24 h after MCAO, for 5 consecutive days.

And also 20 μM miR-132 mimic, miR-132 inhibitor, and miR-132 negative control (Gene Pharma, China) were transfected into primary neurons with Lipofectamine 3000 (Invitrogen, CA, United States) in antibiotic-free Opti-MEM I reduced serum medium, according to the manufacture’s protocol, after 24 h transfection, the primary neurons were exposed to oxygen glucose deprivation (OGD) (Zhou et al., 2017).

### Neurological Evaluation and Infarct Size Assessment

Seven days after reperfusion, an 18-point neurological scoring system based on the method of Garcia et al. (1995) was chosen to assess the neurological deficits by a blinded observer. Then, rats (*n* = 8 for each group) were decapitated. 2-mm thick coronal sections from the brain were obtained by a rat brain matrix and stained with 2% 2, 3, and 5-triphenyltetrazolium chloride (TTC, Sigma-Aldrich) at 37°C for 20 min, and fixed with 4% paraformaldehyde for 24 h. The slices were photographed (FUJIFILM XT1) and the infarct volume was evaluated by imaging software (Adobe Photoshop CC 2015), which was in accordance with the following equation: relative infarct size = (contralateral area–ipsilateral non-infarct area)/contralateral area.

### Neurobehavioral Assessments

Neurobehavioral tests were performed and assessed at baseline and on different days post-stroke by blinded experienced testers. Baseline performance was determined at one day before ischemia. The following behavioral assessments were used in the present study: 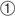 the rotarod test, 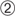 limb placement test, 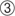 body swing test, 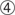 measurement of forelimb placing. The rotarod test (van den Berg et al., 2016) was used to evaluate the motor performances of the rats. The time spent walking on the rotarod without falling was measured twice per animal. The interval between each trial was 15 min. The mean time from two trials was calculated for each rat. The limb placement test (Liu et al., 2006), a composite of motor, sensory, reflex and balance tests, was most commonly used neurological scoring system in animal studies of focal cerebral ischemia. The body swing test (Ingberg et al., 2015) was performed to assess the focal sensorimotor deficits, which was performed without any interfering objects in the immediate surroundings. The proportion of left-side swing was calculated. The measurement of forelimb placing was also performed as previously described (Schallert et al., 2000), which tested sensorimotor/proprioceptive capacity of rats. The percent of unsuccessful contralateral forelimb placing responses was determined.

### Primary Neuronal Culture and OGD Model

Primary neurons were cultured as previously described (Deng et al., 2016). Briefly, neurons were isolated from cerebral cortex of 18-day-old SD rat embryos, washed with D-Hank’s solution three times under sterile conditions, and seeded at a density of 1 × 10^5^ cells/cm^2^ on plates coated with poly-L-lysine (50 mg/mL) (Sigma, United States). The cells were cultured in Neurobasal medium (Gibco, Invitrogen Corp., United States) supplemented with 2% B27, 1% glutamine, and 1% penicillin/streptomycin (Sigma, United States), at 37°C in a humidified incubator with 5% CO_2_, and used after 3 days *in vitro*.

The OGD model was used as previously described (Zhou et al., 2017). Briefly, the cells were incubated in Neurobasal medium that lacked glucose in a humidified incubator with 5% CO_2_ and 95% N_2_ at 37°C for 1 h. Subsequently, primary neurons were returned to original medium and incubated at 37°C under normoxic conditions.

### Dual-Luciferase Reporter Assay

The luciferase complexes were constructed by ligating oligonucleotides containing the wild-type or mutated putative target sites of the rat SOX2 3′-untranslated region (UTR) into the multi-cloning site of the luciferase reporter vector (Genecreat, China). 293T cells were transfected by Lipofectamine 2000 (Invitrogen, United States) according to the manufacturer’s instructions. At 48 h after transfection, the firefly/Renilla luciferase activities were measured using the Dual-Luciferase reporter assay (Promega, United States). The result was used to quantify the miR-132 interaction with the 3′UTR of target gene.

### Immunofluorescence Staining

At 72 h after OGD, neurons were fixed in 4% paraformaldehyde and permeabilized with 5% normal fetal bovine serum in PBS containing 0.1% Triton X-100 for 1 h at room temperature. The primary antibodies including mouse monoclonal anti-(MAP2, NF200, and TAU) antibody (1:500, Millipore, Temecula, CA, United States), and rabbit monoclonal anti-SOX2 antibody (1:100, Abcam, Cambridge, United Kingdom), were incubated with the neurons overnight at 4 C. After the neurons were subjected to three 5-min washes with PBS, they were incubated with FITC-labeled goat anti-mouse IgG (1:2000, Molecular Probes, United States), and Alexa Fluor 594-conjugated anti-rabbit IgG (1:1000, Molecular Probes) for 2 h at room temperature. Finally, they were counterstained with DAPI for 10 min and subjected to another three 10-min washes with PBS. The neurons and neurite length were examined and images were captured using an Olympus BX-60 fluorescence microscope (Olympus Corp., Shinjuku, Tokyo, Japan). Neurite length was measured as the intensity of staining for MAP2/Tau/NF200. MAP2 was abundantly expressed in dendrites and was used as dendrite marker (Harada et al., 2002), TAU was used as axon marker given its abundant expression in axons (Llorens-Martin et al., 2012), and NF200 was used as nerve fiber marker (Schumacher et al., 1999). The average lengths of the longest neurites from three independent experiments were analyzed as previously described (Deng et al., 2014).

### Quantitative Real-Time-Polymerase Chain Reaction (qRT-PCR)

The expression of miR-132 *in vivo* was tested by qRT-PCR analysis. The total RNA was isolated from each sample using RNAiso Plus (TaKaRa) according to a standard protocol and subsequently quantified. cDNAs were prepared using SuperScript III reverse transcriptase (Invitrogen) according to the manufacturer’s instructions. Each sample was tested in triplicate. Primers for miR-132 (GenePharma, Shanghai, China) had the following sequences: ACACTCCAGCTGGGACCGTGGCTTTCGATTGT. The expression of mature miRNAs was measured using Maxima SYBR Green qPCR Master Mix (Fermentas, Burlington, Canada) and the StepOne detection system (Applied Biosystems, Foster City, CA) according to the manufacturer’s instructions, with U6 as an internal control. The relative expression was calculated using the comparative threshold cycle (C_t_) normalized by subtracting the reference gene U6 C_t_ value, which provided the ΔC_t_ value. The relative expression level between treatments was then calculated using the following equation: relative gene expression = 2^-(Δ*Ctsample*-Δ*Ctcontrol*)^ (Liu et al., 2012).

### Western Blotting

To determine the expression of SOX2 in the neurons *in vitro* and *in vivo*, Western blotting analyses were performed after lipofection. The total protein from each group was acquired using an extraction kit (KeyGEN, Nanjing, China) on ice. Primary antibodies were as follows: goat monoclonal anti-SOX2 (1:1000, Abcam, Cambridge, United Kingdom) and rabbit polyclonal anti-Tubulin (1:1000; Santa Cruz Biotechnology, Santa Cruz, CA, United States), which was used as a loading control. Bands were detected using horseradish peroxidase-conjugated secondary antibody (1:5000; Santa Cruz Biotechnology), and immunoreactivity was visualized using an enhanced chemiluminescence kit.

### Statistical Analysis

GraphPad prism 6.0 was used to perform all statistical analyses. All values were presented as mean ± SEM by one-way or two-way analysis of variance (ANOVA). If the ANOVA was significant, then the data were analyzed by pairwise multiple comparison procedures using Tukey’s multiple comparison test. Type I error was defined as <0.5, and the significance was set at a probability *p* < 0.05.

## Results

### EA Treatment Alleviated Brain Injury and Up-Regulated miR-132 Expression in Ischemic Penumbra After Stroke

**FIGURE 1:**
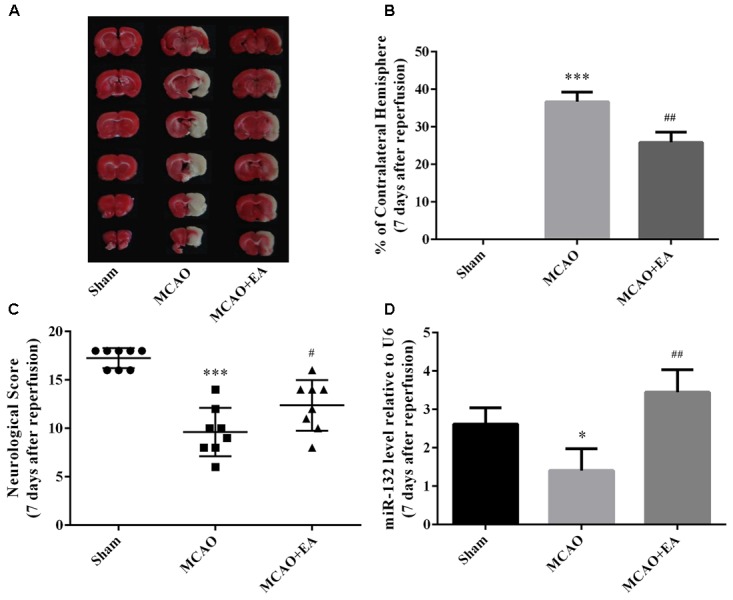
EA treatment alleviated brain injury and up-regulated miR-132 expression in ischemic penumbra after stroke. **(A)** 2, 3, and 5-triphenyltetrazolium chloride (TTC) staining was used to measure infarct volume in coronal brain sections from Sham, MCAO, and MCAO+EA-treated rats at 7 days after reperfusion. **(B)** Bar graph showed the infarct volume in coronal brain sections in each group. **(C)** Neurological score was used to assess recovery of neural function by EA treatment after stroke. **(D)** Bar graph showed the relative expression of miR-132 in the ischemic penumbra in each group. Data are expressed as means ± SEM (*n* = 8 per group); ^∗^*p* < 0.05 vs. Sham, ^∗∗∗^*p* < 0.001 vs. Sham; ^#^*p* < 0.05 vs. MCAO, ^##^*p* < 0.01 vs. MCAO.

To validate the protective effect of EA against cerebral I/R injury, we applied EA to rats after MCAO surgery and then assessed the infarct volume and neurological scores seven days after reperfusion. Consistent with our previous studies (Deng et al., 2016; Zhou et al., 2017), EA treatment significantly reduced the infarct volumes (*p* < 0.01, Figures 1A,B) and improved the neurological scores of MCAO treated rats (*p* < 0.05, Figure 1C). Moreover, miR-132 was down-regulated in MCAO group compared with sham group (*p* < 0.05, Figure 1D), and EA treatment up-regulated the expression of miR-132 in the penumbra of rat brain (*p* < 0.01, Figure 1D). These results indicated that EA exerted neuroprotective effects and reversed the effects of MCAO on the expression of miR-132 in the rat MCAO model.

### Up-Regulation of miR-132 Promoted Neuronal Growth After OGD Injury

**FIGURE 2:**
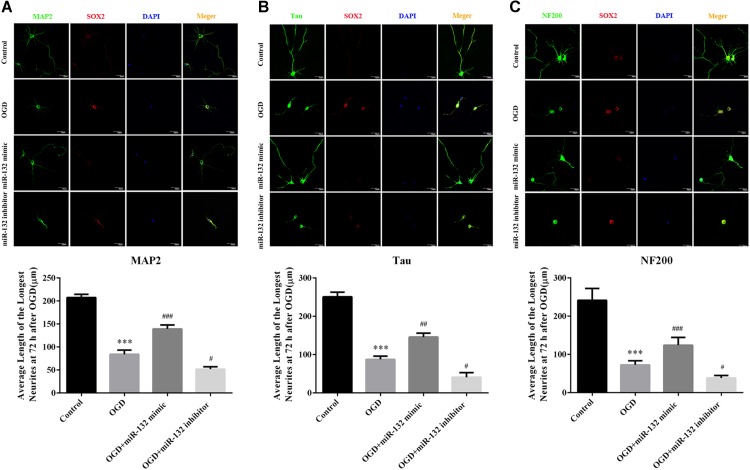
Up-regulation of miR-132 promoted neuronal growth after OGD injury. **(A–C)** Immunofluorescence staining was used to measure neurite outgrowth presented by special marker staining at 72 h after OGD injury. Representative triple-label immunofluorescence staining was performed with MAP2 (green), NF200 (green), Tau (green), SOX2 (red), and DAPI (blue), and the average length of the longest neurite at 72 h after OGD injury were assessed by statistical analysis, respectively. Data are expressed as means ± SEM (*n* = 6 per group); ^∗∗∗^*p* < 0.001 vs. Control; ^#^*p* < 0.05 vs. OGD; ^##^*p* < 0.01 vs. OGD; ^###^*p* < 0.001 vs. OGD.

To further explore the function of miR-132 in ischemic brain injury, we transfected miR-132 mimic or inhibitor into primary neurons before OGD. Then, immunofluorescence staining, which involved representative triple-label staining with MAP2, NF200, and Tau, was used to assess the nerve growth (Figure 2). ANOVA showed a difference among the treatment groups. [*F*(3,20) = 60.37, *p* < 0.001, Figure 2A; *F*(3,20) = 126.2, *p* < 0.001, Figure 2B; *F*(3,20) = 87.45, *p* < 0.001, Figure 2C]. Tukey’s multiple comparison test showed that the longest dendrite length was obviously reduced in OGD group compared with the control group (*p* < 0.001, Figure 2A; *p* < 0.001, Figure 2B; *p* < 0.001, Figure 2C), and the reduction in neuronal growth caused by OGD was almost completely restored by miR-132 mimic (*p* < 0.001, Figure 2A; *p* < 0.01, Figure 2B; *p* < 0.001, Figure 2C). As expected, after miR-132 inhibitor transfection, the dendrite length was further reduced in OGD + miR-132 inhibitor group compared with the OGD group (*p* < 0.05, Figure 2A; *p* < 0.05, Figure 2B; *p* < 0.05, Figure 2C). Additionally, immunofluorescence staining showed that the fluorescence intensity of SOX2 was increased after OGD, which was reduced after miR-132 mimic transfection and was further increased after miR-132 inhibitor transfection. These findings suggested that miR-132 up-regulation facilitated neurite growth after OGD injury and SOX2 might be involved in this process.

### MiR-132 Targeted the Expression of SOX2 *in vitro*

**FIGURE 3:**
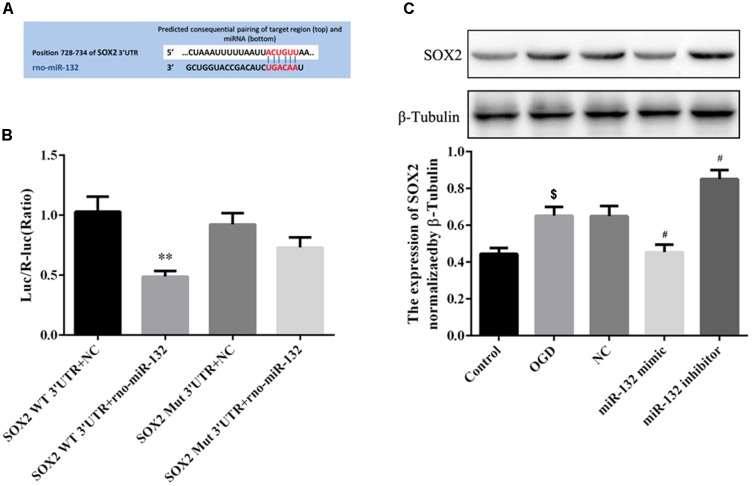
MiR-132 targeted the expression of SOX2 *in vitro*. **(A)** SOX2 was predicted as a target gene of miR-132. **(B)** Dual-Luciferase reporter assay containing the SOX2 wild-type (WT) 3’-UTR or a SOX2 mutant (Mut) 3’-UTR of the SOX2 gene was co-transfected with miR-132 or an empty vector into HEK293T cells, and luciferase activity was assayed. **(C)** Representative Western blotting bands showed SOX2 expression in neurons after transfections with miR-132 mimic and miR-132 inhibitor. Bar graph showed the SOX2 expression levels relative to β-tubulin. Data are expressed as means ± SEM (*n* = 6 per group); ^∗∗^*p* < 0.01 vs. SOX2 WT3’ -UTR + NC, ^$^*p* < 0.05 vs. Control; ^#^*p* < 0.05 vs. OGD.

To explore the potential protective mechanisms of miR-132, we used miRNA database to identify putative miRNA targets and found that SOX2 was a potential target of miR-132 (Figure 3A). Dual-luciferase reporter assay was used to verify whether miR-132 targeted the expression of SOX2. Co-transfection of miR-132 with wild-type SOX2 3′UTR reporter construct significantly inhibited the luciferase activity in 293T cells, while mutation of this target site did not show this change (*p* < 0.01, Figure 3B). These results indicated that SOX2 was a specific target of miR-132.

To further observe the effect of miR-132 on the expression of SOX2, we transfected miR-132 mimic or inhibitor into primary neurons. Western blotting showed that SOX2 expression was significantly decreased in miR-132 mimic group compared with the OGD group (*p* < 0.05, Figure 3C), while the miR-132 inhibitor caused an increase in SOX2 expression (*p* < 0.05, Figure 3C). These results directly demonstrated that miR-132 negatively targeted regulation of SOX2.

### Suppression of SOX2 by miR-132 After Stroke Was Involved in the Neuroprotective Effect of EA Treatment

**FIGURE 4:**
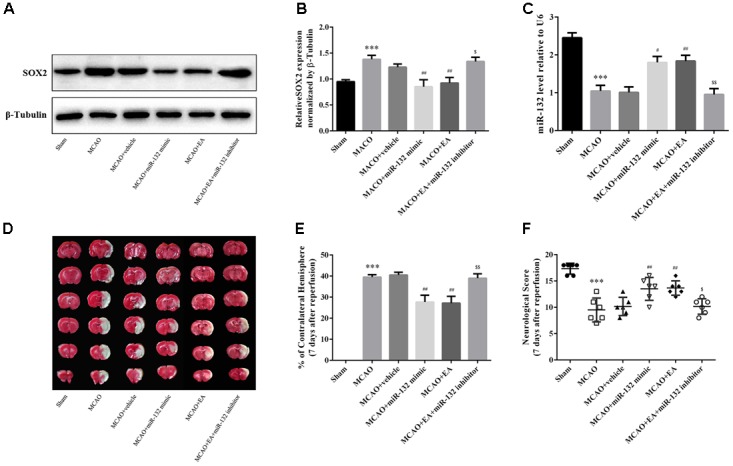
Suppression of SOX2 by miR-132 after stroke was involved in the neuroprotective effect of EA treatment. **(A)** The expression of SOX2 was detected by western blotting in each group at 7 days after MCAO. **(B)** Bar graph showed the SOX2 expression level relative to β-tubulin. **(C)** miR-132 expression level relative to U6 was also evaluated at 7 days after reperfusion. **(D)** TTC staining was used to evaluate infarct volume after miR-132 mimic or miR-132 inhibitor injection, MCAO and/or EA treatment. **(E)** Bar graph showed the infarct volume in coronal brain sections in each group. **(F)** Garcia scoring system was used to assess recovery of neural function after stroke by miR-132 mimic or miR-132 inhibitor injection, MCAO and/or EA treatment. Data are expressed as means ± SEM (*n* = 6 per group); ^∗∗∗^*p* < 0.001 vs. sham; ^#^*p* < 0.05, ^##^*p* < 0.01 vs. MACO; ^$^*p* < 0.05, ^$$^*p* < 0.01 vs. MACO+EA.

To determine the protective effect of suppression of SOX2 by miR-132 in EA treatment against cerebral I/R injury, intracerebroventricular injection of miR-132 mimic or inhibitor was administered 24 h after reperfusion with or without EA treatment, for 5 consecutive days. The assessment indicators were performed 7 days after reperfusion. The results indicated that EA treatment or miR-132 mimic transfection obviously reduced the level of SOX2 in ischemic penumbra (*p* < 0.001 or *p* < 0.01, Figures 4A,B). The miR-132 inhibitor obviously reversed the reduction of SOX2 by EA, suggesting that SOX2 may be the key for protective effect of miR-132 in EA treatment against cerebral I/R injury. We also assessed the miR-132 level in ischemic penumbra by miR-132 mimic or inhibitor. qRT-PCR analysis showed that miR-132 level was obviously reduced in MCAO group compared with the sham group (*p* < 0.001, Figure 4C). However, EA treatment or miR-132 mimic transfection significantly increased the miR-132 level in ischemic penumbra of MCAO-induced rats (*p* < 0.05 or *p* < 0.01, Figure 4C). Interestingly, miR-132 inhibitor significantly reversed the increased level of miR-132 by EA treatment (*p* < 0.01, Figure 4C). Furthermore, we assessed the infarct volume and neurological scores 7 days after reperfusion. The results showed that the infarct volume was obviously reduced in MCAO + EA and MCAO + miR-132 mimic groups compared with the MCAO group, while the neurological scores was improved in MCAO + EA and MCAO + miR-132 mimic groups compared with the MCAO group (*p* < 0.05 or *p* < 0.01, Figures 4D–F). However, these effects were obviously reversed by miR-132 inhibitor transfection (*p* < 0.05 or *p* < 0.01, Figures 4D,E). These results further suggested that miR-132 was involved in EA neuroprotective effect by targeting inhibition of SOX2.

### Over-Expression of miR-132 Enhanced Neurobehavioral Functional Recovery After Stroke *in vivo*

**FIGURE 5:**
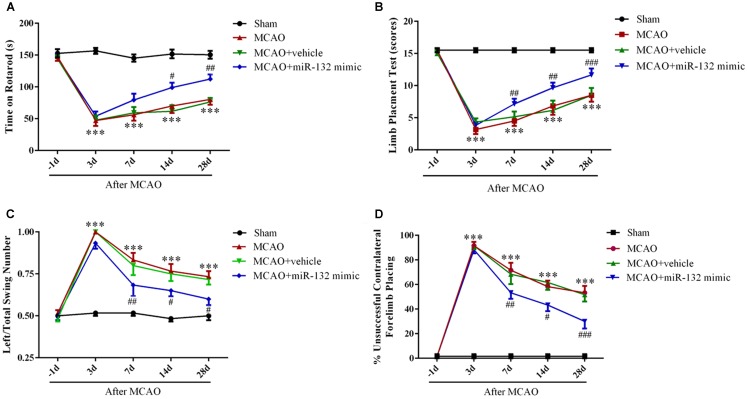
Over-expression of miR-132 enhanced neurobehavioral functional recovery after stroke *in vivo*. The Rotarod test **(A)**, limb placement test **(B)**, left/total swing numbers **(C)**, and unsuccessful contralateral forelimb placement **(D)** were performed at baseline and 3, 7, 14, and 28 days after MCAO and miR-132 mimic injection; ^∗∗∗^*p* < 0.001 vs. Sham; *^#^p* < 0.05, ^##^*p* < 0.01, ^###^*p* < 0.001 vs. MCAO.

To further verify whether up-regulation of miR-132 is beneficial for neurobehavioral functional rehabilitation, intracerebroventricular injection of miR-132 mimic was followed by the rotarod test, limb placement test, body swing test, and measurement of forelimb placing. The results suggested that the time on rotarod and limb placement scores were obviously reduced at 3, 7,14, and 28 days after MCAO in MCAO group compared with the sham group (*p* < 0.001, Figures 5A,B), while miR-132 mimic transfection significantly reversed the reduction of the time on rotarod at 14 and 28 days after MCAO (*p* < 0.05, *p* < 0.01, Figure 5A) and the reduction of limb placement scores at 7, 14, and 28 days after MCAO (*p* < 0.01, *p* < 0.001, Figure 5B). Conversely, the left/total swing numbers and the percent of unsuccessful contralateral forelimb placement were significantly increased at 3, 7, 14, and 28 days after MCAO in MCAO group compared with the sham group (*p* < 0.001, Figures 5C,D). As expected, the increase by MCAO at 7, 14, and 28 days was significantly reversed by miR-132 mimic (*p* < 0.05, *p* < 0.01 or *p* < 0.001, Figures 5C,D). These results suggested that miR-132 enhanced neurobehavioral functional recovery after stroke.

### Down-Regulation of miR-132 Reversed the Effect of Improved Rehabilitation by EA After Stroke

**FIGURE 6:**
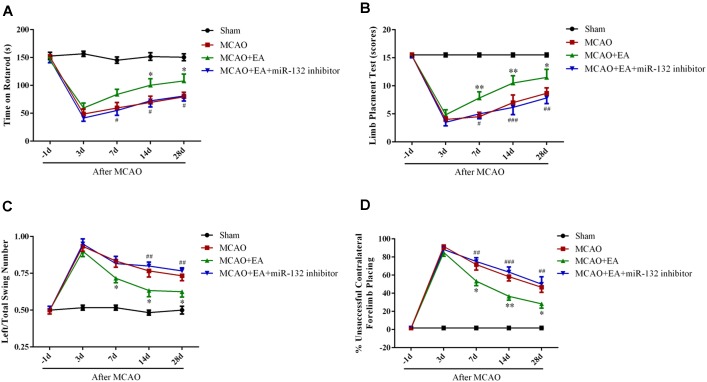
Down-regulation of miR-132 reversed the effect of improved rehabilitation by EA after stroke. The Rotarod test **(A)**, limb placement test **(B)**, left/total swing numbers **(C)**, and unsuccessful contralateral forelimb placement **(D)** were performed at baseline and 3, 7, 14, and 28 days after MCAO; ^∗^*p* < 0.05, ^∗∗^*p* < 0.01 vs. MCAO; *^#^p* < 0.05, ^##^*p* < 0.01, ^###^*P* < 0.001 vs. MCAO+EA.

To further confirm that EA exerts neuroprotective effects by up-regulation of miR-132, miR-132 inhibitor was intracerebroventricularly injected after EA treatment, followed by the rotarod test, limb placement test, body swing test, and measurement of forelimb placing. The results showed that the time of on rotarod at 14 and 28 days after MCAO (*p* < 0.05, Figure 6A) and the scores of the limb placement at 7,14, and 28 days after MCAO were increased in MCAO+EA group compared with the MCAO group (*p* < 0.05, *p* < 0.01, Figure 6B); however, miR-132 inhibitor transfection significantly reversed EA effect at 7, 14, and 28 days after MCAO (*p* < 0.05, *p* < 0.01, and *p* < 0.001, Figures 6A,B). Conversely, the left/total swing numbers and the percent of unsuccessful contralateral forelimb placement at 7, 14, and 28 days after MCAO were significantly reduced in MCAO + EA group compared with the MCAO group (*p* < 0.05, Figure 6C; *p* < 0.05 or *p* < 0.01, Figure 6D). As expected, the reduction effect at 7, 14, and 28 days after MCAO by EA was significantly reversed by miR-132 inhibitor transfection (*p* < 0.01, Figure 6C; *p* < 0.01 or *p* < 0.001, Figure 6D). These results further suggested that EA had neuroprotective effect and improved neurobehavioral functional rehabilitation by up-regulation of miR-132 after stroke.

## Discussion

Stroke remains a major healthcare challenge despite the increasing availability of acute thrombolytic interventions such as thrombolysis and endovascular treatment strategies (Lackland et al., 2014). The long-term disability has become common sequela in the aftermath of stroke, which costs the United States economy $34 billion annually, including the costs of healthcare services, medications, and lost productivity (Mozaffarian et al., 2016). Therefore, effective therapies targeting to improve neurological recovery among survivors of stroke is crucial. Acupuncture or EA has shown positive effects against brain damage after stroke (Cao et al., 2016; Deng et al., 2016; Jiang et al., 2016). Similarly, EA treatment is found to significantly reduce infarct volume and improve the neurological scores. Motor impairments are the most common neurobehavioral functional deficit after stroke (Li, 2017). Moreover, spasticity and motor recovery are both related to neural plasticity and plastic reorganization after stroke (Li, 2017). In the present study, neurobehavioral assessments (motor function and coordination ability by the rotarod and swing test, sensorimotor/proprioceptive capacity by the limb placement and the rate of unsuccessful contralateral forelimb placement test) after MCAO showed that EA improved neurobehavioral functional rehabilitation after stroke. However, the underlying mechanism remains unknown.

Study has indicated that miRNAs are involved in the process of ischemia injury in brain (Dickson et al., 2010). In our previous study (Deng et al., 2016), miRNA microarray analysis showed that many miRNAs, including miR-132, were significantly changed in the cortex of the penumbra after MCAO and EA treatment. In the present study, qRT-PCR showed that the expression of miR-132 increased in MCAO-induced rats after EA treatment.

To further explore the effect of miR-132 during brain ischemia injury and EA treatment, miR-132 mimic and inhibitor, respectively, packaged into liposome were injected into the intracerebroventricular region of rat brains after MCAO and primary neurons which were isolated from neonatal rats before OGD. The results demonstrated that overexpression of miR-132 by miR-132 mimic reduced infarct volume and alleviated neurological deficit after stroke, similar to the effect of EA on MCAO. Conversely, inhibition of miR-132 significantly reversed the improvement after EA treatment on MCAO. Previous studies have indicated that after ischemia, neurofunctional recovery was associated with neural plasticity including axonal sprouting and remodeling, neurite outgrowth, increases in synaptic proteins neurabin and neurexin, and the formation of new projections from CST (Ruscher et al., 2011; Liu et al., 2013). MiRNAs have emerged as key regulators for neuroplasticity after stroke (Kassis et al., 2017). In the present study, immunofluorescence staining was used to assess the change in neuronal growth by transfection of miR-132 mimic or inhibitor in primary neurons. MiR-132 mimic increased, while miR-132 inhibitor reduced the length of dendritic protrusions labeled by MAP2, axonal protrusions labeled by Tau, and nerve fibers labeled by NF200 in OGD-induced 293T cell. These results indicated that up-regulation of miR-132 promoted neural plasticity. Taken together, the above results suggested that up-regulation of miR-132 could induce neuroprotection *in vitro* and *in vivo*, and down-regulation of miR-132 partly reversed the neuroprotection after EA treatment.

MicroRNAs exert biological effects by targeting numerous downstream genes, multiple complex protein functions and pathways. Mature miRNAs recognize the 3′UTR regions of their target messenger RNAs (mRNAs) through Watson-Crick base pairing (Bushati and Cohen, 2007). In this study, we examined a possible relationship between miR-132 and SOX2 mRNA by Dual-luciferase reporter assay, and investigated whether putative SOX2 was the target of miR-132 by bioinformatics algorithms (TargetScan, miRBase). Neural cells with high SOX2 expression are incapable of entering in either of two differentiation pathways, neurogenesis or gliogenesis. Moreover, SOX2 overexpression reduces the number of mature MAP2-positive neurons (Klajn et al., 2014). Another study also indicates that increased SOX2 does not promote adult hippocampal neural progenitor cell proliferation (Peltier et al., 2011). In the present study, we found that overexpression of miR-132 down-regulated the expression of SOX2, while inhibition of miR-132 up-regulated the expression of SOX2 in the MCAO-induced rats and OGD-induced 293T cells. In addition, the level of SOX2 in MCAO after EA was reversed after miR-132 inhibitor transfection. Our results also showed that miR-132 mimic reduced the fluorescence intensity of SOX2 and miR-132 inhibitor increased fluorescence intensity of SOX2 by immunofluorescence staining, which were consistent with previous protein detection results. These results confirmed that up-regulation of SOX2 by miR-132 inhibitor reduced the length of neurons. However, the complex and multiple biological functions of SOX2 need to be further studied. The above results confirm that the expression of miR-132 correlates inversely with the expression of SOX2 in the rat brain penumbra, which suggests a potential regulatory mechanism for the neuroprotection induced by EA treatment.

**FIGURE 7:**
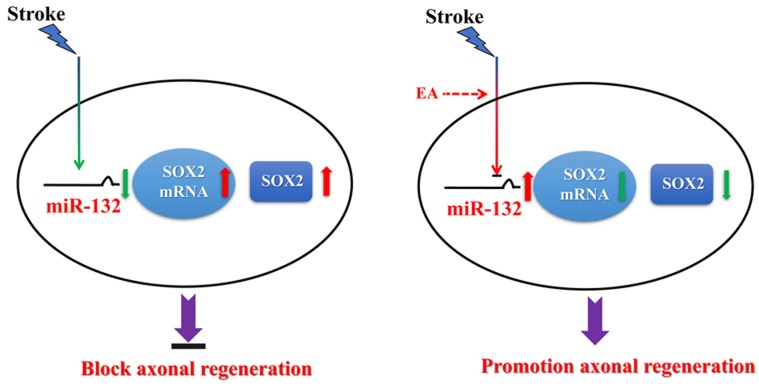
Hypothetical model depicting mechanisms underlying the therapeutic effects of miR-132 by EA against ischemic stroke. EA up-regulates miR-132 level and then miR-132 promotes neurite outgrowth and enhances neurobehavioral functional recovery after stroke by targeting inhibition of SOX2 mechanism.

In summary, the results indicate that EA up-regulates miR-132, which targets inhibition of SOX2 transcriptional expression, to promote axonal regeneration and enhance neurobehavioral functional recovery after stroke, suggesting that miR-132 and SOX2 act as key regulators of axonal regeneration after cerebral I/R injury (Figure 7). Our results provide evidence that EA treatment induces epigenetic changes to regulate its targets, such as the miR-132/SOX2 axis, which may be a novel mechanism underlying EA treatment for ischemic stroke.

## Author Contributions

XZ and FB had full access to all the data in the study, take responsibility for the integrity of the data and the accuracy of the data analysis, and drafted the manuscript. QW, XZ, and FB conceptualized and designed the study. XZ, FB, EZ, DZ, TJ, and HZ performed the experiment and acquired, analyzed, or interpreted the data. QW critically revised the manuscript for important intellectual content.

## Conflict of Interest Statement

The authors declare that the research was conducted in the absence of any commercial or financial relationships that could be construed as a potential conflict of interest.
